# Effects of complexity and seasonality on backward bifurcation in vector–host models

**DOI:** 10.1098/rsos.171971

**Published:** 2018-02-28

**Authors:** Shakir Bilal, Edwin Michael

**Affiliations:** Department of Biological Sciences, University of Notre Dame, Notre Dame, IN 46556, USA

**Keywords:** complexity, seasonality, backward bifurcation and control, malaria, dengue, West Nile virus

## Abstract

We study implications of complexity and seasonality in vector–host epidemiological models exhibiting backward bifurcation. Vector–host diseases represent complex infection systems that can vary in the transmission processes and population stages involved in disease progression. Seasonal fluctuations in external forcing factors can also interact in a complex way with internal host factors to govern the transmission dynamics. In backward bifurcation, the insufficiency of *R*_0_ < 1 for predicting the stability of the disease-free equilibrium (DFE) state arises due to existence of bistability (coexisting DFE and endemic equilibria) for a range of *R*_0_ values below one. Here we report that this region of bistability decreases with increasing complexity of vector-borne disease transmission as well as with increasing seasonality strength. The decreases in the bistability region are accompanied by a reduced force of infection acting on primary hosts. As a consequence, we show counterintuitively that a more complex vector-borne disease may be easier to control in settings of high seasonality.

## 1. Introduction

Predicting the elimination/extinction of an infectious disease from a population is an important question that confronts policy makers in the event of a disease outbreak. Microparasitic vector borne diseases such as West Nile virus (WNV), chikungunya, dengue, malaria and Zika differ in complexity, are prevalent or are emergent in different parts of the world [1–10]. The transmission cycles of these diseases are maintained in various hosts such as birds, humans and other animals, with the degree of complexity differing between diseases in terms of the stages involved in the progression of disease in each host population, and specifics of the structure and processes governing the transmission process between hosts [11,12].

The essential features of the transmission dynamics of microparasitic diseases can be studied via compartmental models, whereby the populations of hosts and vectors are divided into distinct classes or compartments, each defining the state of an individual with respect to the stage of disease progression, e.g. susceptible, exposed, infectious, recovered hosts [11]. Increasing the number of compartments and transmission structures in such a system increases realism, but also behavioural complexity in these models [13,14]. For these models, the basic reproduction ratio (*R*_0_), defined as the number of secondary cases produced by a single infected case, can serve as an indicator of disease endemicity in the population. In the simplest case (e.g. the susceptible-infected-recovered (SIR) model), the condition *R*_0_ = 1 defines a bifurcation (or critical) point, whereby when *R*_0 _> 1 a stable infected equilibrium emerges and establishes, whereas when it is less than unity the disease-free equilibrium (DFE) emerges and is generally globally stable [11].

By contrast, recently it has been shown that compartmental models may describe a scenario in which a turning point of the infected equilibrium may exist in a region where all equilibrium states are positive and *R*_0_ < 1 [15–18]. This induces multiple stable equilibria and disruption of the global stability of the disease-free equilibrium, with the result that instead of converging globally to the disease free state when *R*_0 _< 1, the solution may approach an infected state that coexists with the disease-free state. Several disease models have been investigated for the existence of this phenomenon in the literature [1,5,15,19–23], and compartmental models developed for vector-borne diseases, including WNV [1,19,24], dengue [21], malaria [5], and other arboviral diseases [22] have also been shown to exhibit backward bifurcation and thus violation of the *R*_0_ = 1 threshold condition separating diseases endemicity and extinction [23].

Several mechanisms leading to backward bifurcation have been proposed, such as partially effective vaccination programmes [25,26], social differences in susceptibles [17], nonlinear incidences [15], the structure of interactions among multi-groups [27–29], and multiple stages of infection [30]. However two features appear important: a nonlinear incidence rate due to saturation effects [13,31]; and asymmetry in the susceptibility of hosts to infection [19], including in the death rates of susceptible and infected hosts. The latter disease induced increase in death rates among infected hosts along with a mass action force of infection has been shown to be a particularly important driving factor for the occurrence of backward bifurcation in WNV, dengue, malaria and other arboviral models [5,19,20].

Seasonal variation in reported cases of infectious diseases, ranging from childhood diseases (e.g. measles, diphtheria and chickenpox) to infections affecting all ages like influenza, cholera and vector borne diseases (e.g. WNV, malaria, dengue), is common across temperate and tropical geographies [32–34]. In compartmental models for malaria, it has been shown that periodic changes in mosquito populations can reduce the basic reproductive ratio [35–37]. Similarly, the recurrence of WNV in many previously affected areas and incursion into new geographical areas is also, in most cases, related to seasonal effects on vector population dynamics [24]. There is a growing body of literature [11,12,32–34,38] investigating the role of external drivers (e.g. rainfall, temperature) responsible for seasonal fluctuations in disease incidence, but few studies exist that have investigated the impact of these important external drivers in diseases suspected to show backward bifurcation [39].

In this paper, we investigate the dynamics of a general host susceptible-exposed-infective-temporarily recovered (SEIRS)–vector SEI model, and tune it to study the effect that complexity (in terms of structure, topological structure of interactions and parameters) may have on the phenomenon of backward bifurcation. We also study the influence of seasonality on the phenomenon using the model. To ground the analysis, we investigated the impact of both factors using parameter regimes derived from the literature for WNV, dengue and malaria.

The subsequent sections are organized as follows. In the upcoming section we first describe the SEIRS–SEI–SEIRS model and derive a quadratic equation for endemic equilibrium where a dead-end host is absent. In the results section, we evaluate and present the conditions for backward bifurcation to exist in the presence of seasonality in our disease systems, and compare them with the non-seasonal case. We then investigate the effect of disease complexity by adding compartments beginning from the $\text{SI}-M_{S}M_{I}$ stage of the model (see below), and follow this by quantifying the effect of including a dead-end host. We end by discussing the likely occurrence, extent, and the factors that underlie backward bifurcation in our disease systems, and the implications they have for disease control.

## 2. Material and methods

### 2.1. General representation of compartmental models

A typical compartmental model describes infection progression between host classes representing susceptibles, exposed, infectives and recovered individuals [11,12]. During an emerging outbreak, all individuals are susceptible; once infected they can remain dormant (exposed) for some time before infecting others or they immediately become infectives and start infecting others. Infected individuals may (i) recover, (ii) die due to infection, or (iii) become susceptible again.

In general, in this system, a heterogeneous population can thus be grouped into *N* homogeneous compartments. We can rearrange the compartments such that the first *m* of them correspond to the infected (including symptomatic and asymptomatic) while the rest correspond to uninfected classes. The time evolution of the state of the population {*x_i_*} has the general form:

2.1 $$\frac{\text{d}x_{i}}{\text{d}t}=F_{i}-V_{i},$$

where $F_{i}$ represents input rate of newly infected individuals in the *i*th compartment and $V_{i}=V_{i}^{-}-V_{i}^{+}$ with $V_{i}^{\pm }$ being the transfer in/out of the compartment *i* by all other means. Writing the compartmental model in the form of equation (2.1) facilitates the calculation of the basic reproductive ratio *R*_0_ for complex models involving multiple hosts or vectors [40] as shown in the electronic supplementary material, appendix. This framework also allows development of a hierarchical set of specific models of increasing or decreasing complexity. Below we begin by describing a maximal (SEIRS–SEI–SEIRS) model for these systems, reflecting the dynamics of transmission represented by WNV. We then show how complexity may be reduced to capture the dynamics of dengue and malaria transmission.

### 2.2. The seasonal SEIRS–SEI–SEIRS model

**Figure 1.**
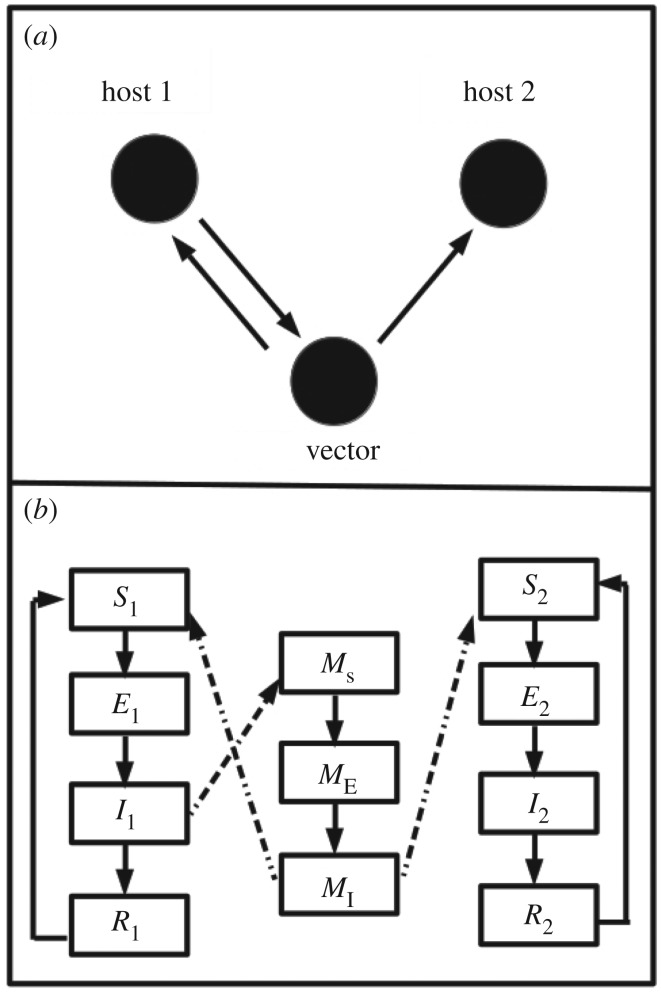
A one-vector–two-host system where the second host is a dead end. This is equivalent to a network of three nodes where the vector node infects the two host nodes but gets infected by only one host node. (a) The host--vector interaction network. (b) Flow chart for the host--vector interaction network.

A one-vector–two-host system where the second host is a dead end is characterized by a network of three nodes where the vector node infects the two host nodes but gets infected by only one host node, as shown in figure 1. The mutually infecting vector–host nodes form the primary cycle of the disease. Here infection progression in each host node is described by a susceptible (S), exposed (E), infective (I), temporarily recovered (R) (SEIRS) model while the vector node is described by a susceptible (*M*_S_), exposed (*M*_E_), infective (*M*_I_) (SEI) model assuming that vectors do not recover from the disease. The equations for the model are as follows:

2.2 $$\begin{array}{rl} & \;\begin{array}{rl} \frac{\text{d}S_{1}}{\text{d}t} & \;=\Pi_{H1}-\lambda_{H1}S_{1}-\mu_{H1}S_{1}+\alpha_{H1}R_{1}\\ \frac{\text{d}E_{1}}{\text{d}t} & \;=\lambda_{H1}S_{1}-(\mu_{H1}+\sigma_{H1})E_{1}\\ \frac{\text{d}I_{1}}{\text{d}t} & \;=\sigma_{H1}E_{1}-(\tau_{H1}+d_{H1}+\mu_{H1})I_{1}\\ \frac{\text{d}R_{1}}{\text{d}t} & \;=\tau_{H1}I_{1}-\mu_{H1}R_{1}-\alpha_{H1}R_{1} \end{array}\} \end{array}$$

2.3 $$\begin{array}{r} \;\begin{array}{rl} \frac{\text{d}S_{2}}{\text{d}t} & \;=\Pi_{H2}-\lambda_{H2}S_{2}-\mu_{H2}S_{2}+\alpha_{H2}R_{2}\\ \frac{\text{d}E_{2}}{\text{d}t} & \;=\lambda_{H2}S_{2}-(\mu_{H2}-\sigma_{H2})E_{2}\\ \frac{\text{d}I_{2}}{\text{d}t} & \;=\sigma_{H2}E_{2}-(\tau_{H2}+d_{H2}+\mu_{H2})I_{2}\\ \frac{\text{d}R_{2}}{\text{d}t} & \;=\tau_{H2}I_{2}-\mu_{H2}R_{2}-\alpha_{H2}R_{2} \end{array}\} \end{array}$$

2.4 $$\begin{array}{r} \;\begin{array}{rl} \frac{\text{d}M_{S}}{\text{d}t} & \;=\Pi_{M}-\lambda_{M}M_{S}-\mu_{M}M_{S}\\ \frac{\text{d}M_{E}}{\text{d}t} & \;=\lambda_{M}M_{S}-(\mu_{M}-\sigma_{M})M_{E}\\ \frac{\text{d}M_{I}}{\text{d}t} & \;=\sigma_{H1}M_{E}-(d_{M}+\mu_{M})M_{I} \end{array}\} \end{array}$$

2.5 $$\begin{array}{r} \lambda_{H1}=\frac{b_{1}\beta_{H1}}{N_{H1}}(\eta_{M}M_{E}+M_{I}) \end{array}$$

2.6 $$\begin{array}{r} \lambda_{H2}=\frac{b_{2}\beta_{H2}}{N_{H2}}(\eta_{M}M_{E}+M_{I}) \end{array}$$

2.7 $$\begin{array}{r} \lambda_{M}=\frac{b_{1}\beta_{1}}{N_{H1}}(\eta_{H1}E_{1}+I_{1}) \end{array}$$

2.8 $$\begin{array}{r} \;\begin{array}{rl} b_{1} & \;=\frac{bN_{H1}}{\left(N_{H1}+N_{H2}\right)}\\ \text{and}\;b_{2} & \;=\frac{bN_{H2}}{\left(N_{H1}+N_{H2}\right)} \end{array}\}, \end{array}$$

where the state variables $\left(S_{1},E_{1},I_{1},R_{1}\right)$ correspond to the primary host-1, $\left(S_{2},E_{2},I_{2},R_{2}\right)$ correspond to secondary host-2, $\left(M_{S},M_{E},M_{I}\right)$ correspond to vectors, $\lambda_{H1},\lambda_{H2},\lambda_{M}$ are force of infection on host 1, host 2, and vectors respectively. The total number of mosquito bites *b* are divided between hosts 1 and host 2 and are given by *b*_1_ and *b*_2_. The definition of other parameters are given in table 1. Seasonal variations are introduced by a sinusoidal mosquito birth rate $\Pi_{M}(t)$ as discussed in the electronic supplementary material, appendix. In all our later calculations we will assume $\eta_{M}=\eta_{H1}=d_{M}=0$. The model equations (2.2)–(2.4) have a disease free equilibrium given by:

2.9 $$\begin{array}{rl} E_{0} & \;=(S_{1}^{*},E_{1}^{*},I_{1}^{*},R_{1,}^{*}S_{2}^{*},E_{2}^{*},I_{2}^{*},R_{2}^{*},M_{S}^{*},M_{E}^{*},M_{I}^{*})\\ & \;=\left(\frac{\Pi_{H1}}{\mu_{H1}},0,0,0\frac{\Pi_{H2}}{\mu_{H2}},0,0,0,\frac{\Pi_{M}}{\mu_{M}},0,0\right). \end{array}$$

Definition of *R*_0_ in presence of seasonality is not as straightforward. The linear operator method [41,42] can be used to obtain an accurate estimate of *R*_0_; however in most cases it involves numerical solutions. Since the total population of mosquitoes is governed by $\text{d}N_{M}\text{/d}t=\Pi_{M}(t)-\mu_{M}N$, at equilibrium this is equivalent to a sinusoidally varying population and opens up the methods of Bacar [37] to be applied for calculating *R*_0_ as a function of seasonality strength *ε*, i.e. *R*_0_(*ε*). The method of Bacar [37] is based on using a combination of the survival function approach and Fourier analysis. It is approximate but provides a deeper analytical understanding of the effects of seasonality. We give a summary of both these procedures in electronic supplementary material, appendix, but use the later method unless specified otherwise. The expression for *R*_0_(*ε*) is obtained as:

2.10 $$\begin{array}{rl} R_{0}(\varepsilon ) & =R_{0}\left(1-\frac{\varepsilon^{2}}{2}G\right) \end{array}$$

2.11 $$\begin{array}{rl} G & =\frac{Q_{1}Q_{2}Q_{3}Q_{4}g_{1}}{\Omega^{2}{g_{1}}^{2}+{g_{2}}^{2}} \end{array}$$

2.12 $$\begin{array}{rl} g_{1} & =\left[-\Omega^{2}+\overset{3}{\underset{l=1}{\sum }}\overset{4}{\underset{m=l+1}{\sum }}Q_{l}Q_{m}\right]\\ g_{2} & =\left(\Omega^{2}\overset{4}{\underset{l=1}{\sum }}Q_{l}-Q_{1}Q_{2}(Q_{3}+Q_{4})-Q_{3}Q_{4}(Q_{1}+Q_{2})\right)\\ R_{0} & =\frac{b_{1}^{2}\beta_{1}\beta_{2}\prod_{M}\mu_{H1}\sigma_{H1}\sigma_{M}}{\prod_{H}\mu_{M}Q_{1}Q_{2}Q_{3}Q_{4}} \end{array}$$

where $Q_{1}=\mu_{H1}+\sigma_{H1},Q_{2}=\tau_{H1}+d_{H1}+\mu_{H1},Q_{3}=\mu_{M}+\sigma_{M},Q_{4}=\mu_{M}+d_{M}.$ *R*_0_ is the basic reproduction ratio, in the absence of seasonality, as obtained using the survival function approach [43], and Ω=2π/T is the oscillation frequency, *T*  =  365 is the period of oscillation in days and the function *G* is obtained using the methods of Bacar [37]. For the parameters of diseases considered here, equation (2.10) leads to the condition $R_{0}(\varepsilon )<R_{0}$.

**Table 1.** Parameters and their interpretation in the full SEIRS–SEI model. A blank (–) unit means the parameter is dimensionless.

parameter	interpretation	units
*b*	average mosquito biting rate	—
*b*_1_	mosquito biting rate on host 1	day^−1^
*b*_2_	mosquito biting rate on host 2	day^−1^
$\beta_{1}$	transmission probability host 1 to mosquito	—
$\beta_{H1}$	transmission probability mosquito to host 1	—
$\beta_{H2}$	transmission probability mosquito to host 2	—
$\Pi_{M}$	mosquito birth rate	day^−1^
$\mu_{M}$	mosquito death rate	day^−1^
$\Pi_{H1}$	host 1 birth rate	day^−1^
$\Pi_{H2}$	host 2 birth rate	day^−1^
$\mu_{H1}$	host 1 death rate	day^−1^
$\mu_{H2}$	host 2 death rate	day^−1^
*d*_M_	disease induced death rate mosquito	day^−1^
$d_{H1}$	disease induced death rate in hosts 1	day^−1^
$d_{H2}$	disease induced death rate in hosts 2	day^−1^
$\frac{1}{\sigma_{M}}$	exposed period mosquito	day
$\frac{1}{\sigma_{H1}}$	exposed period in hosts 1	day
$\frac{1}{\sigma_{H2}}$	exposed period in hosts 2	day
$\tau_{H1}$	host 1 recovery rate	day^−1^
$\tau_{H2}$	host 2 recovery rate	day^−1^
$\alpha_{H1}$	host 1 loss of immunity rate	day^−1^
$\alpha_{H2}$	host 2 loss of immunity rate	day^−1^
$\eta_{M}$	modification parameter mosquito	—
$\eta_{H1}$	modification parameter host 1	—

The endemic equilibrium of the equations (2.2)–(2.4) is given by:

2.13 $$\begin{array}{rl} & \text{host}\;\;\;\;\;1-\left\{\begin{array}{rlrl} & S_{1}^{*}=\frac{\Pi_{H1}}{\left(\lambda_{H1}+\mu_{H1}-f1\right)}, & & \;\;E_{1}^{*}=\frac{\lambda_{H1}\Pi_{H1}}{Q_{1}(\lambda_{H1}+\mu_{H1}-f1)}\\ & I_{1}^{*}=\frac{\sigma_{H1}\lambda_{H1}\Pi_{H1}}{Q_{1}Q_{2}(\lambda_{H1}+\mu_{H1}-f1)}, & & \;\;R_{1}^{*}=\frac{\tau_{H1}\sigma_{H1}\lambda_{H1}\Pi_{H1}}{\left(\mu_{H1}+\alpha_{H1})Q_{1}Q_{2}(\lambda_{H1}+\mu_{H1}-f1\right)} \end{array}\right\} \end{array}$$

2.14 $$\begin{array}{rl} & \text{host}\;\;\;\;2-\left\{\begin{array}{rlrl} & S_{2}^{*}=\frac{\Pi_{H1}}{\left(\lambda_{H1}+\mu_{H1}-f2\right)}, & & \;\;E_{2}^{*}=\frac{\lambda_{H1}\Pi_{H1}}{Q_{1}(\lambda_{H1}+\mu_{H1}-f2)}\\ & I_{2}^{*}=\frac{\sigma_{H1}\lambda_{H1}\Pi_{H1}}{Q_{1}Q_{2}(\lambda_{H1}+\mu_{H1}-f2)}, & & \;\;R_{2}^{*}=\frac{\tau_{H1}\sigma_{H1}\lambda_{H1}\Pi_{H1}}{\left(\mu_{H1}+\alpha_{H1})Q_{1}Q_{2}(\lambda_{H1}+\mu_{H1}-f2\right)} \end{array}\right\} \end{array}$$

2.15 $$\begin{array}{rl} & \text{and}\;\;\text{mosquito}-\left\{\begin{array}{c} M_{S}^{*}=\frac{\Pi_{H1}}{\left(\lambda_{H1}+\mu_{H1}-f1\right)},\;M_{E}^{*}=\frac{\lambda_{H1}\Pi_{H1}}{Q_{1}(\lambda_{H1}+\mu_{H1}-f1)}\\ M_{I}^{*}=\frac{\sigma_{H1}\lambda_{H1}\Pi_{H1}}{Q_{1}Q_{2}(\lambda_{H1}+\mu_{H1}-f1)} \end{array}\right\}, \end{array}$$

where $Q_{5}=\mu_{H2}+\sigma_{H2},Q_{6}=\tau_{H2}+d_{H2}+\mu_{H2}$ and

$$\begin{array}{rl} f1 & \;=\frac{\alpha_{H1}\tau_{H1}\lambda_{H1}\sigma_{H1}}{Q_{1}Q_{2}(\mu_{H1}+\alpha_{H1})}\\ f2 & \;=\frac{\alpha_{H2}\tau_{H2}\lambda_{H2}\sigma_{H2}}{Q_{5}Q_{6}(\mu_{H2}+\alpha_{H2})}. \end{array}$$

For the case of small secondary host population *N*_H2_ ≈ 0, then inserting equation (2.13) and equation (2.15) into the forces of infection equations (2.5) and (2.7), it is easy to see that *λ*_H1_ satisfies the quadratic equation

2.16 $$\begin{array}{rl} & a_{0}{\lambda_{H1}}^{2}+b_{0}\lambda_{H1}+c_{0}=0 \end{array}$$

2.17 $$\begin{array}{rl} & a_{0}=\prod_{H1}Q_{3}Q_{4}[\left(\mu_{H1}+\alpha_{H1})Q_{2}+\sigma_{H1}(\mu_{H1}+\alpha_{H1}\tau_{H1}\right)][b_{1}\beta_{1}(\eta_{H1}Q_{2}+\sigma_{H1})(\mu_{H1}+\alpha_{H1})\\ & \;+(\mu_{H1}+\alpha_{H1})\mu_{M}(Q_{2}+\sigma_{H1})+\mu_{M}\tau_{H1}\sigma_{H1}] \end{array}$$

2.18 b0=∏H1Q1Q2Q3Q4{b1β1(μH1+αH1)(ηH1Q2+σH1)+μMQ1Q2(μH1+αH1)−αH1τH1σH1μH12((μH1+αH1)σH1+τH1σH1+Q2(μH1+αH1))−R0(ε)Q1Q2(μH1+αH1)−αH1τH1σH1μH1

2.19 $$\begin{array}{rl} & \text{and}\;\;c_{0}={Q_{1}}^{2}{Q_{2}}^{2}Q_{3}Q_{4}\prod_{H1}{\left(\mu_{H1}+\tau_{H1}\right)}^{2}\mu_{M}(1-{R_{0}}^{2}(\varepsilon )). \end{array}$$

It is reasonable to assume that a quadratic equation of the above type continues to exist even when $N_{H2}$ is finite and is confirmed in numerical simulations, which we discuss below.

### 2.3. Complexity

Complexity of a model can be broadly divided into two categories:

1. The number of distinct host/vector species affecting the disease spread. This corresponds to distinct nodes as shown in figure 1*a*.
2. Each node is represented by a compartmental model: the complexity can thus also increase as the number of compartments and/or parameters governing the transfer from one compartment to another is increased.

To illustrate the second form of complexity we take the example of just the primary host–vector cycle ($N_{H2}=0$): setting $\left(\sigma_{\left\{\text{M},\text{H}1\right\}}\to \infty ,\alpha_{H1}=0,\tau_{H1}=0\right)$ implies that there are no exposed and recovered classes and hence the $\text{SI}-M_{S}M_{I}$ model is obtained (WNV [1,18]), setting $\left(\sigma_{\left\{\text{M},\text{H}1\right\}}\to \infty ,\alpha_{H1}\neq 0,\tau_{H1}\neq 0\right)$ the $\text{SIRS}-M_{S}M_{I}$ model (malaria [5]) is obtained, and $\alpha_{H1}=0$ reduces equation (2.2) to $\text{SEIR}-M_{S}M_{E}M_{I}$ model (dengue one serotype [20,21]). The first type of complexity is introduced by additional host/vector species and in our case we add a dead-end host. The presence of dead-end hosts such as humans and other mammals in the vicinity of birds has relevance for WNV [24].

Although transmission complexity can be introduced in a variety of ways, e.g. nonlinear transmission rate arising due to saturation effects, in this work we consider a linear transmission rate to minimize system nonlinearity, allowing us to investigate complexity in terms of increasing compartments and hosts.

### 2.4. Sensitivity index

We investigated the effect of parameters on the backward bifurcation critical point *R*_c_ using sensitivity analysis. The sensitivity index ($\gamma_{p}^{u}$) of a variable *u* with respect to the parameter *p* is formally defined as the ratio of relative change in a variable *u* to the relative change in the parameter *p* [44]:

2.20 $$\gamma_{p}^{u}=\frac{\partial u}{\partial p}\times \frac{p}{u}.$$

## 3. Results

In light of equations (2.16)–(2.19), we can state the equivalent of Theorem 1 in (3.1) that the primary host–vector (SEIRS–SEI) model has:

1. a unique endemic equilibrium if $R_{0}(\varepsilon )>1$ (as *c*_0 _< 0)
2. a unique endemic equilibrium if *b*_0 _< 0 and *c*_0 _= 0 or ${b_{0}}^{2}-4a_{0}c_{0}=0$
3. two endemic equilibria if *c*_0 _> 0, *b*_0 _< 0 and ${b_{0}}^{2}-4a_{0}c_{0}>0$
4. no endemic equilibrium otherwise.

Using the condition ${b_{0}}^{2}-4a_{0}c_{0}=0$ the critical value of $R_{0}(\varepsilon )$ for backward bifurcation, denoted by $R_{c}(\varepsilon )$, is then obtained as:

3.1 $$R_{c}(\varepsilon )=\frac{1}{2(1-(\varepsilon^{2}\text{/}2)G)}\left(A+\sqrt{A^{2}-4B}\right),$$

where *A* and *B* are functions of parameters except the seasonality strength (*ε*), see electronic supplementary material, appendix. The range $R_{c}\leq R\leq 1$ is the backward bifurcation region.

**Figure 2.**
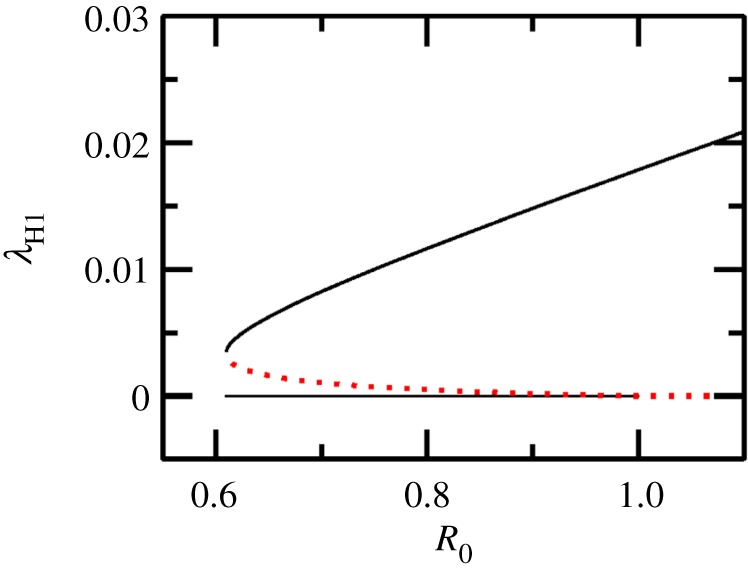
Bifurcation diagram as a function of *R*_0_ in the absence of seasonality. Here the top black and red curves are the force of infection $\lambda_{H1}$ corresponding to the stable and unstable endemic states of host 1 when *R*_0_ ≤ 1. The black horizontal line is the disease-free equilibrium (DFE). At *R*_0_ ≥ 1 the DFE becomes unstable and only one stable endemic state exists. The parameters for WNV from table 3 were used in the SEIRS–SEI–SEIRS model, with no secondary hosts, to generate this figure.

**Figure 3.**
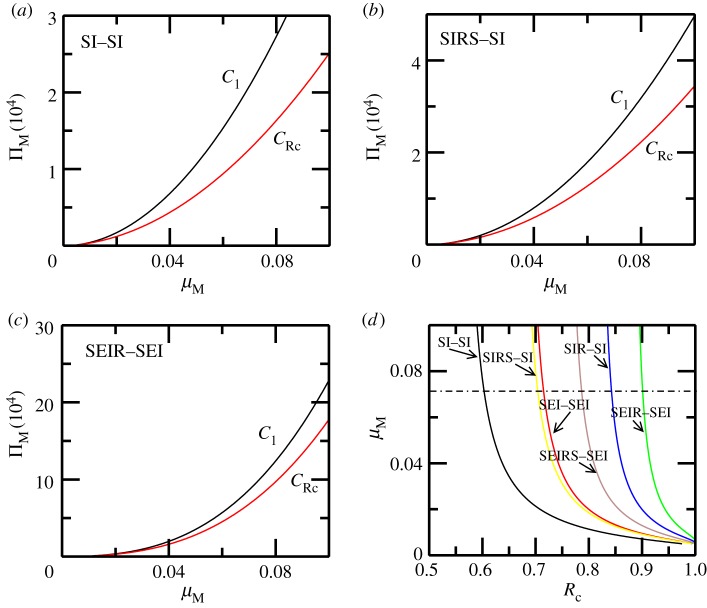
The model (without seasonality) and effects of complexity. The backward bifurcation region in the $\Pi_{M}-\mu_{M}$ space is sandwiched between $C_{1}$ and $C_{\mathrm{Rc}}$ curves which correspond to $R_{0}=1$ and $R_{0}=R_{c}$ respectively. The plots in (*a–c*) show that as *R*_c_ increases due to increasing complexity it also shifts the *C*_1_ and $C_{\mathrm{Rc}}$ curves thus allowing for better control by varying the mosquito birth/death rates. In (*d*) the shift of *R*_c_ curve is shown in the $R_{c}-\mu_{M}$ space as more compartments are added to the model. Here the WNV model parameters (table 3) were used in the $\text{SI}-M_{S}M_{I}$ approximation model and $\sigma_{H1}=0.016,\sigma_{M}=0.016,\alpha_{H1}=0.001,\tau_{H1}=0.001$ were used to change the complexity.

**Figure 4.**
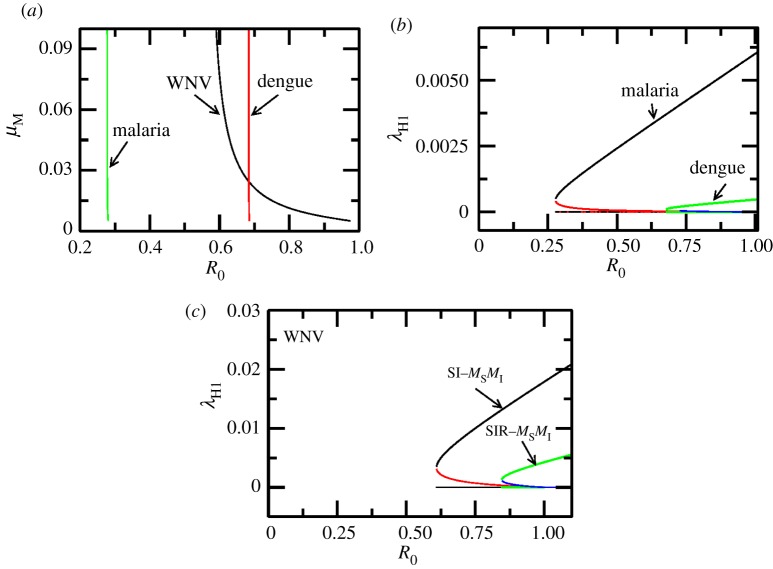
(*a*) The backward bifurcation boundary *R*_c_ in the $R_{0}-\mu_{M}$ space for the model parameters corresponding to WNV (black), dengue (red) and malaria (green) from table 3. Although *R*_c_ varies with $\mu_{M}$ in all three cases the range of variation is largest for WNV parameters. The backward bifurcation region is largest for malaria followed by WNV and dengue when $\mu_{M}=1/16.0$ (assuming a mosquito lifespan of 16 days). (*b*,*c*) Bifurcation diagrams showing stable and unstable branches of (*b*) force of infection $\lambda_{H1}$ as a function of *R*_0_ for malaria and dengue and (*c*) force of infection for WNV model with $\text{SI}-M_{S}M_{I}$ along with an $\text{SIR}-M_{S}M_{I}$ model with a small recovery rate $\tau_{H1}=0.001$ (cf. Figure 3*d*). We plotted dengue and malaria in the same graph because of similarity in model dimension and parameters in contrast to WNV. Even though the WNV shows large force of infection as compared to malaria and dengue, this may be because no latent period was considered for WNV along with incomparable natural and disease-induced host death rates. However, the general principle that a larger force of infection increases the backward bifurcation region in both (*b*) and (*c*) can be seen to hold.

Bifurcation diagrams in this paper are obtained using the Newton–Raphson root finding procedure for stable and unstable fixed points of a dynamical system [45] (unless mentioned otherwise). In the absence of seasonality, i.e. *ε* = 0, a bifurcation diagram in figure 2 for the $\text{SI}-M_{S}M_{I}$ approximation of the model shows that the endemic state coexists with the DFE (black horizontal line) in the backward bifurcation region $R_{c}\leq R\leq 1$. Clearly, since $R_{0}<1$ is not a sufficient criterion for disease elimination, it is instructive to look at the backward bifurcation region in the $\Pi_{M}-\mu_{M}$ space, since $\Pi_{M}$ and $\mu_{M}$ can be changed by mosquito control measures. In the $\Pi_{M}-\mu_{M}$ parameter space the area between the curves *C*_1_ and *C*_RC_ represents the backward bifurcation region as shown in figure 3*a* for the $\text{SI}-M_{S}M_{I}$ approximation of the model. As the complexity of the model is increased by bringing into play more compartments/parameters, the backward bifurcation region is altered in significant ways: for example, a simple inclusion of temporary immunity shifts the curves *C*_1_ and *C*_RC_ so that a larger value of $\Pi_{M}$ (for a given $\mu_{M}$) is required to be in the backward bifurcation region. Activating an exposed/latent state increases this threshold further, as shown in figure 3*b*,*c*. The shift in *R*_c_ with addition of more compartments is easily seen in the $R_{0}-\mu_{M}$ space where it translates into an increase in *R*_c_ as shown in figure 3*d*. In general, a compartment representing the delay of infectious state or a temporary (permanent) immunity in hosts increases the threshold *R*_c_ w.r.t the basic $\text{SI}-M_{S}M_{I}$ model. The sensitivity indices $\gamma_{p}^{R_{c}}$ (see equation (2.20)) of *R*_c_ w.r.t parameters (*p*) are tabulated in table 2. A positive (or negative) value of $\gamma_{p}^{R_{c}}$ indicates that an increase in parameter *p* also increases (or decreases) *R*_c_. According to table 2 the parameters of the model clearly form two groups based on whether they increase or decrease the value of *R*_c_, assuming an increase in these parameters. Increasing the values in the first group $\left(b_{1},\beta_{1},\mu_{H1},\varepsilon ,\tau_{H1}\right)$ increases while increasing the values of the second group $\left(d_{H1},\mu_{M},\sigma_{H1},\alpha_{H1}\right)$ decreases the threshold *R*_c_. Counterintuitively, $\left(\beta_{H1},\Pi_{H2}\right)$ do not change *R*_c_, while a weakly positive but negligible dependence on $\sigma_{M}$ is observed. The parameter regimes of WNV, dengue, malaria (table 3) produce different values of *R*_c_ in the $R_{0}-\mu_{M}$ space, as shown in figure 4, therefore the relative change in *R*_c_ is calculated from their respective base values of *R*_c_. As an example, for WNV $R_{c}∼0.609$ and $\gamma_{\mu_{H1}}^{R_{c}}∼0.6415$, which means that a 6.4% change in $\mu_{H1}$ produces a 10% change in the value of *R*_c_.

**Table 2.** Sensitivity index $\gamma_{p}^{R_{c}}$ w.r.t model parameters (*p*) for WNV, dengue and malaria. We took ε=0.3 while calculating $\gamma_{\varepsilon }^{R_{c}}$ and ε=0 for the rest $\gamma_{p}^{R_{c}}$.

parameter (*p*)	WNV	dengue	malaria
*b*_1_	0.08394292488	0.0002338511190	0.0009617256570
$\beta_{H1}$	0	0	0
$\beta_{1}$	0.08394292485	0.0002338511190	0.0009617256566
$\mu_{M}$	−0.08394292425	−0.0002338534960	−0.0009617250206
$\Pi_{H1}$	0	0	0
$\mu_{H1}$	0.6414893380	0.0009251279255	0.1790822307
$d_{H1}$	−0.6415417466	−0.5655065196	−0.8441519805
$\sigma_{M}$	0	0	0
$\sigma_{H1}$	−0.3345086117 × 10^−5^	−0.0006105349835	−0.006315525812
$\tau_{H1}$	0	0.5651919267	0.8427761814
$\alpha_{H1}$	0	0	−0.1713909052
ε	0.002159482349	0.0004596099794	0.00007150530087

**Table 3.** Model parameters for WNV, dengue and malaria. Model parameters for WNV, dengue and malaria are based on the following references: [1,19], ([21], E Michael 2015, unpublished work) and [44]. The units of parameters are given in table 1.

parameter	WNV	dengue	malaria
*b*	0.1	0.315	0.42
$\beta_{1}$	0.16	0.9	0.64
$\beta_{H1}$	0.88	0.8	0.08
$\beta_{H2}$	0.88	0	0
$\Pi_{M}$	variable	variable	variable
$\mu_{M}$	0.0625	0.0625	0.0625
$\Pi_{H1}$	1000	1000	1000
$\Pi_{H2}$	variable	0	0
$\mu_{H1}$	0.001	$\frac{1}{70\times 365}$	$\frac{1}{70\times 365}$
$\mu_{H2}$	$\frac{1}{70\times 365}$	—	—
$d_{M}$	0	0	0
$d_{H1}$	0.005	0.99	0.99
$d_{H2}$	10^−5^	—	—
$\sigma_{M}$	1000	0.0904	0.0909
$\sigma_{H1}$	1000	0.1671	0.0714
$\sigma_{H2}$	0.0714	—	—
$\tau_{H1}$	0	0.274	0.1
$\tau_{H2}$	0.0714	—	—
$\alpha_{H1}$	0	0	0.00001
$\alpha_{H2}$	0	—	—
$\eta_{M}$	0	0	0
$\eta_{H1}$	0	0	0

**Figure 5.**
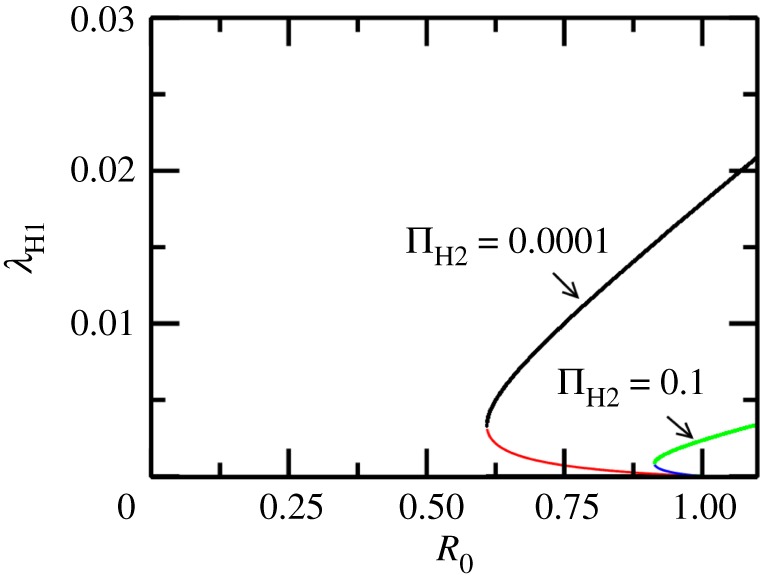
WNV model with humans as dead-end hosts. A bifurcation diagram showing stable (black and green) and unstable (red and blue) branches of the force of infection $\lambda_{H1}$ as a function of dead-end host population (governed by the birth rate $\Pi_{H2}$). It shows that the force of infection reduces as the number of bites on the dead-end hosts increases (see equations (2.5)--(2.8)).

When the dead-end host is also integrated into the model, as the number of secondary hosts (given by $N_{H2}=\Pi_{H2}\text{/}\mu_{H2}$) is increased the backward bifurcation region decreases, as shown in figure 5 for the WNV model parameters listed in table 3. This is because involving secondary dead-end hosts will lead to a reduction in the overall force of infection (figure 5).

**Figure 6.**
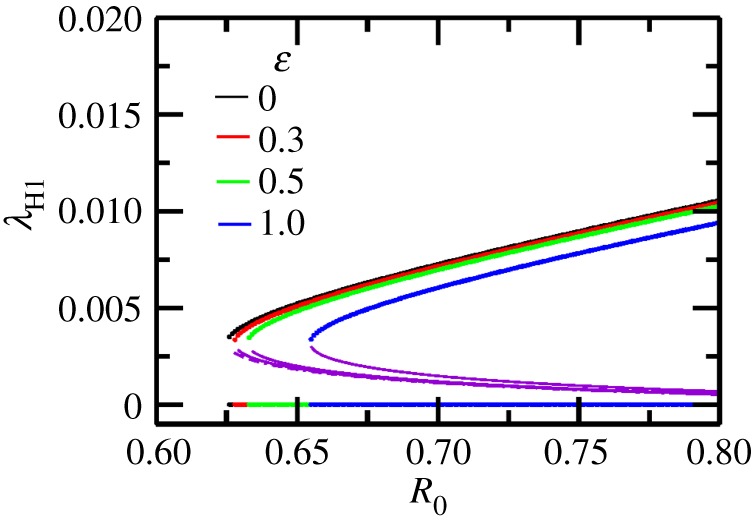
A bifurcation diagram showing stable (colours as labelled) and unstable (violet) branches of the force of infection $\lambda_{H1}$ as a function of *ε*. It shows that the force of infection reduces, resulting in increase of *R*_c_, as *ε* is increased. To obtain this figure we use the non-seasonal equations and replace $\Pi_{M0}$ by $\Pi_{M0}(1-(\varepsilon^{2}\text{/}2)G)$ for a given *R*_0_ (equation (2.12)) in the model equation (2.2) and then evaluate the stable and unstable branches using the Newton–Raphson method (see text for justification of this procedure).

When seasonality is switched on (ε>0), it is easy to see from equation (3.1) that the threshold *R*_c_ increases with increasing seasonality strength. A representative bifurcation diagram shown in figure 6 illustrates this result. The bifurcation diagram was obtained by replacing $\Pi_{M0}$ with $\Pi_{M0}(1-(\varepsilon^{2}\text{/}2)G)$ in the nonseasonal equations. This substitution simplifies the calculation of the bifurcation diagram and is justified from the following two observations: (i) *R*_0_ in equation (2.12) (non-seasonal case) then becomes equal to *R*_0_(*ε*) in equation (2.10) (seasonal case), and (ii) a bifurcation diagram using the linear operator method for *R*_0_(*ε*) with the original seasonal term also shows trends similar to figure 6 with increasing seasonality (see electronic supplementary material, figure S1). It also follows, as a consequence of equation (3.1), that the threshold mosquito birth rate is higher in the seasonal case as compared to the non-seasonal case. The sensitivity index $\gamma_{\varepsilon }^{R_{c}}$ in table 2 confirms this result and it also shows that the WNV parameters lead to a stronger change in *R*_c_ when compared to dengue/malaria for the seasonal case.

We also find that the reduced/increased backward bifurcation region for all the cases above is always accompanied by a reduced/increased force of infection on primary hosts $\lambda_{H1}$. This is easily deduced from the bifurcation diagrams in figures 4–6.

## 4. Discussion

The condition *R*_0 _< 1 for stability of DFE breaks down due to the backward bifurcation phenomenon. It has been shown to exist in models of many vector-borne diseases [1,21,24]. However, previous studies ignored the impact of increasing complexity, including seasonality.

In this work we argued that the $\text{SI}-M_{S}M_{I}$ approximation of the model is a basic vector–host model exhibiting backward bifurcation. Then the impact of increasing complexity and seasonality on *R*_c_ was studied. For instance the addition of exposed or immune compartments in hosts changes the $\text{SI}-M_{S}M_{I}$ model to an $\text{SEI}-M_{S}M_{I}$ or $\text{SIR}-M_{S}M_{I}$ model and in both cases the threshold *R*_c_ is found to increase, with the addition of recovereds found to have the stronger effect. On the other hand, if immunity is temporary in the $\text{SIR}-M_{S}M_{I}$ model then the resulting $\text{SIRS}-M_{S}M_{I}$ model has a relatively lower threshold value *R*_c_ though still higher than the original $\text{SI}-M_{S}M_{I}$ model. Therefore, in general, increasing model complexity (by adding delay to the infectious state or a temporary (permanent) immunity in hosts) leads to an increase in the threshold *R*_c_ w.r.t the basic $\text{SIR}-M_{S}M_{I}$ model. Similarly, the addition of a dead-end host in the model also reduces the backward bifurcation region for the simple reason that if mosquitoes bite dead-end hosts more, such bites will result in reducing the overall disease transmission rate.

For the parameter regimes of WNV [1,19], malaria [5,44] and dengue ([21], E Michael 2015, unpublished work) the sensitivity analyses carried out in this study show that the host (bird) disease-induced death rate strongly affects the backward bifurcation region for all diseases with increases in death rates of infecteds under conditions of variable birth rates increasing the backward bifurcation region. However, for the WNV case, these results also show that increasing the natural death of hosts (birds) more markedly increases the threshold *R*_c_. This indicates that culling of hosts (birds) could constitute the major strategy for managing WNV spread in areas where backward bifurcation is possible for this disease. By contrast, because increased immunity will decrease the backward bifurcation regions more for dengue and malaria (table 2), improving the rate of host recovery by vaccination may be a more effective control strategy for these diseases.

Seasonality always reduces the value of *R*_0_ resulting in an increase in the value of *R*_c_; this implies that it is easier to control a vector-borne disease in highly seasonal areas relative to weakly seasonal ones. Based on our results we observe that although the backward bifurcation region decreases in the order of malaria, WNV and dengue, the results from the sensitivity analysis predict that seasonality has stronger effect on WNV followed by dengue and malaria.

In general, however, we note that given the observation that an increase in *R*_c_ values is a consequence of a reduced force of infection acting on primary hosts, this effect could be used to develop a guiding principle for designing complexity-based control programmes for vector-borne diseases. For example, this could involve, given the specificity of the disease, culling of primary hosts such as birds in the case of WNV, adding of secondary hosts in malaria (zooprophylaxis) or vaccination of humans (as proposed for dengue and malaria).

The simple sinusoidal variation in mosquito birth rates studied in this work may not capture the full complexity of seasonality; incorporating the exact form of this factor has been pointed out as a significant element to be considered in disease modelling (see the arguments put forth in [38,46]), and growing empirical evidence indicates that these external forcings (i.e. via rainfall, temperature fluctuations acting on vector populations as well as pathogen development rates) may indeed vary year-to-year [47,48]. However, based on the fact that any oscillatory signal could be expanded in a Fourier series with multiple frequencies, it is reasonable to assume that calculations of *R*_0_(*ε*) reduce to the results of Bacar [37] when all but one frequency has a dominant role.

Incorporating nonlinear transmission rates (depicting saturation effects among others), and variable rather than fixed parameter range also increase the complexity of the models. An investigation of nonlinear forms of the transmission rate, multiple host–vector models, seasonality reflected via multiple frequencies with different amplitudes, and variable parameter ranges to gain a fuller understanding of process complexity in these systems clearly form a task for future work.

## Supplementary Material

Basic Reproductive ratio

## Data accessibility

No data were used or generated during the study. Only numerical simulations were performed based on standard numerical algorithms. The parameters used in the simulation are available in the papers cited.

## References

[1] Bowman C, Gumel AB, Van den Driessche P, Wu J, Zhu H 2005 A mathematical model for assessing control strategies against West Nile virus. Bull. Math. Biol. 67, 1107–1133. (doi:10.1016/j.bulm.2005.01.002) 15998497 10.1016/j.bulm.2005.01.002

[2] Thomas DM, Urena B 2001 A model describing the evolution of West Nile-like encephalitis in New York City. Math. Comput. Model 34, 771–781. (doi:10.1016/S0895-7177(01)00098-X)

[3] Wonham MJ, de-Camino-Beck T, Lewis MA 2004 An epidemiological model for West Nile virus: invasion analysis and control applications. Proc. R. Soc. B 271, 501–507. (doi:10.1098/rspb.2003.2608) 10.1098/rspb.2003.2608 PMC1691622 15129960

[4] Andraud M, Hens N, Marais C, Beutels P 2012 Dynamic epidemiological models for dengue transmission: a systematic review of structural approaches. PLoS ONE 7, e49085 (doi:10.1371/journal.pone.0049085) 23139836 10.1371/journal.pone.0049085 PMC3490912

[5] Tilahun M 2017 Backward bifurcation in SIRS malaria model. arXiv preprint (arXiv:1707.00924).

[6] Mandal S, Sarkar RR, Sinha S 2011 Mathematical models of malaria: a review. Malaria J. 10, 202 (doi:10.1186/1475-2875-10-202) 10.1186/1475-2875-10-202 PMC3162588 21777413

[7] Lee EK, Liu Y, Pietz F 2016 A compartmental model for Zika virus with dynamic human and vector populations. In *AMIA Annual Symposium Proceedings*, pp. 743–752. American Medical Informatics Association.

[8] Li R et al. 2017 Zika virus infections, a review. Radiol. Infect. Dis. 4, 88–93. (doi:10.1016/j.jrid.2017.01.002)

[9] Plourde AR, Bloch EM 2016 A literature review of Zika virus. Emerg. Infect. Dis. 22, 1185 (doi:10.3201/eid2207.151990) 27070380 10.3201/eid2207.151990 PMC4918175

[10] Yakob L, Clements AC 2013 A mathematical model of chikungunya dynamics and control: the major epidemic on Réunion Island. PLoS ONE 8, e57448 (doi:10.1371/journal.pone.0057448) 23554860 10.1371/journal.pone.0057448 PMC3590184

[11] Keeling MJ, Rohani P 2008 Modeling infectious diseases in humans and animals. Princeton, NJ: Princeton University Press.

[12] Anderson RM, May RM, Anderson B. 1992 Infectious diseases of humans: dynamics and control. Wiley Online Library.

[13] Zhang W, Yu P, Wahl LM 2015 Backward bifurcation underlies rich dynamics in simple disease models. arXiv preprint (arXiv:1504.05260).

[14] Aguiar M, Kooi BW, Rocha F, Ghaffari P, Stollenwerk N 2013 How much complexity is needed to describe the fluctuations observed in dengue hemorrhagic fever incidence data? Ecol. Complexity 16, 31–40. (doi:10.1016/j.ecocom.2012.09.001)

[15] van den Driessche P, Watmough J 2000 A simple SIS epidemic model with a backward bifurcation. J. Math. Biol. 40, 525–540. (doi:10.1007/s002850000032) 10945647 10.1007/s002850000032

[16] Romero DM, Kribs-Zaleta CM, Mubayi A, Orbe C 2011 An epidemiological approach to the spread of political third parties. Discrete and Continuous Dynamical Systems-Series B 15, 707–738. (doi:10.3934/dcdsb.2011.15.707)

[17] Hadeler KP, Van den Driessche P 1997 Backward bifurcation in epidemic control. Math. Biosci. 146, 15–35. (doi:10.1016/S0025-5564(97)00027-8) 9357292 10.1016/S0025-5564(97)00027-8

[18] Hadeler KP, Castillo-Chvez C 1995 A core group model for disease transmission. Math. Biosci. 128, 41–55. (doi:10.1016/0025-5564(94)00066-9) 7606144 10.1016/0025-5564(94)00066-9

[19] Jiang J, Qiu Z, Wu J, Zhu H 2009 Threshold conditions for West Nile virus outbreaks. Bull. Math. Biol. 71, 627–647. (doi:10.1007/s11538-008-9374-6) 19101771 10.1007/s11538-008-9374-6

[20] Gumel AB 2012 Causes of backward bifurcations in some epidemiological models. J. Math. Anal. Appl. 395, 355–365. (doi:10.1016/j.jmaa.2012.04.077)

[21] Garba SM, Gumel AB, Bakar MA 2008 Backward bifurcations in dengue transmission dynamics. Math. Biosci. 215, 11–25. (doi:10.1016/j.mbs.2008.05.002) 18573507 10.1016/j.mbs.2008.05.002

[22] Abboubakar H, Kamgang JC, Tieudjo D 2016 Backward bifurcation and control in transmission dynamics of arboviral diseases. Math. Biosci. 278, 100–129. (doi:10.1016/j.mbs.2016.06.002) 27321192 10.1016/j.mbs.2016.06.002

[23] Li J, Blakeley D, Smith RJ 2011 The failure of *R*_0_. Comput. Math. Methods Med. 2011, 527610 (doi:10.1155/2011/527610) 21860658 10.1155/2011/527610 PMC3157160

[24] Blayneh KW, Gumel AB, Lenhart S, Clayton T 2010 Backward bifurcation and optimal control in transmission dynamics of West Nile virus. Bull. Math. Biol. 72, 1006–1028. (doi:10.1007/s11538-009-9480-0) 20054714 10.1007/s11538-009-9480-0

[25] Brauer F 2004 Backward bifurcations in simple vaccination models. J. Math. Anal. Appl. 2982, 418–431. (doi:10.1016/j.jmaa.2004.05.045)

[26] Arino J, McCluskey CC, van den Driessche P 2003 Global results for an epidemic model with vaccination that exhibits backward bifurcation. SIAM J. Appl. Math. 64, 260–276. (doi:10.1137/S0036139902413829)

[27] Castillo-Chavez C, Cooke KL, Huang W, Levin SA. 1989 On the role of long incubation periods in the dynamics of acquired immunodeficiency syndrome (AIDS). In Part 2: multiple group models. Mathematical and statistical approaches to AIDS epidemiology, pp. 200–217. Berlin, Germany: Springer.

[28] Castillo-Chavez C, Cooke K, Huang W, Levin SA 1989 Results on the dynamics for models for the sexual transmission of the human immunodeficiency virus. Appl. Math. Lett. 2, 327–331. (doi:10.1016/0893-9659(89)90080-3)

[29] Huang W, Cooke KL, Castillo-Chavez C 1992 Stability and bifurcation for a multiple-group model for the dynamics of HIV/AIDS transmission. SIAM J. Appl. Math. 52, 835–854. (doi:10.1137/0152047)

[30] Simon CP, Jacquez JA 1992 Reproduction numbers and the stability of equilibria of SI models for heterogeneous populations. SIAM J. Appl. Math. 52, 541–576. (doi:10.1137/0152030)

[31] Korobeinikov A, Maini PK 2005 Non-linear incidence and stability of infectious disease models. Math. Med. Biol. 22, 113–128. (doi:10.1093/imammb/dqi001) 15778334 10.1093/imammb/dqi001

[32] Altizer S, Dobson A, Hosseini P, Hudson P, Pascual M, Rohani P 2006 Seasonality and the dynamics of infectious diseases. Ecol. Lett. 9, 467–484. (doi:10.1111/j.1461-0248.2005.00879.x) 16623732 10.1111/j.1461-0248.2005.00879.x

[33] Grassly NC, Fraser C 2006 Seasonal infectious disease epidemiology. Proc. R. Soc. B 273, 2541–2550. (doi:10.1098/rspb.2006.3604) 10.1098/rspb.2006.3604 PMC1634916 16959647

[34] Fisman DN 2007 Seasonality of infectious diseases. Annu. Rev. Public Health 28, 127–143. (doi:10.1146/annurev.publhealth.28.021406.144128) 17222079 10.1146/annurev.publhealth.28.021406.144128

[35] Parham PE, Michael E 2011 Outbreak properties of epidemic models: the roles of temporal forcing and stochasticity on pathogen invasion dynamics. J. Theor. Biol. 271, 1–9. (doi:10.1016/j.jtbi.2010.11.015) 21094169 10.1016/j.jtbi.2010.11.015

[36] Parham PE, Michael E 2010 Modeling the effects of weather and climate change on malaria transmission. Environ. Health Perspect. 118, 620 (doi:10.1289/ehp.0901256) 20435552 10.1289/ehp.0901256 PMC2866676

[37] Bacar N 2007 Approximation of the basic reproduction number R_0_ for vector-borne diseases with a periodic vector population. Bull. Math. Biol. 69, 1067–1091. (doi:10.1007/s11538-006-9166-9) 17265121 10.1007/s11538-006-9166-9

[38] Pascual M, Dobson A 2005 Seasonal patterns of infectious diseases. PLoS Med. 2, e5 (doi:10.1371/journal.pmed.0020005) 15696215 10.1371/journal.pmed.0020005 PMC545198

[39] Ngonghala CN, Ngwa GA, Teboh-Ewungkem MI 2012 Periodic oscillations and backward bifurcation in a model for the dynamics of malaria transmission. Math. Biosci. 240, 45–62. (doi:10.1016/j.mbs.2012.06.003) 22732318 10.1016/j.mbs.2012.06.003

[40] Van den Driessche P, Watmough J 2002 Reproduction numbers and sub-threshold endemic equilibria for compartmental models of disease transmission. Math. Biosci. 180, 29–48. (doi:10.1016/S0025-5564(02)00108-6) 12387915 10.1016/s0025-5564(02)00108-6

[41] Wang W, Zhao X 2008 Threshold dynamics for compartmental epidemic models in periodic environments. J. Dyn. Differ. Equations 20, 699–717. (doi:10.1007/s10884-008-9111-8)

[42] Mitchell CD 2016 Reproductive numbers for periodic epidemic systems. PhD thesis, Department of Mathematics, University of Texas at Arlington, TX.

[43] Heffernan JM, Smith RJ, Wahl LM 2005 Perspectives on the basic reproductive ratio. J. R. Soc. Interface 2, 281–293. (doi:10.1098/rsif.2005.0042) 16849186 10.1098/rsif.2005.0042 PMC1578275

[44] Chitnis N, Hyman JM, Cushing JM 2008 Determining important parameters in the spread of malaria through the sensitivity analysis of a mathematical model. Bull. Math. Biol. 70, 1272–1296. (doi:10.1007/s11538-008-9299-0) 18293044 10.1007/s11538-008-9299-0

[45] Parker TS, Chua L 2012 Practical numerical algorithms for chaotic systems. Berlin, Germany: Springer Science & Business Media.

[46] Laneri K, Bhadra A, Ionides EL, Bouma M, Dhiman RC, Yadav RS, Pascual M 2010 Forcing versus feedback: epidemic malaria and monsoon rains in northwest India. PLoS Comput. Biol. 6, e1000898 (doi:10.1371/journal.pcbi.1000898) 20824122 10.1371/journal.pcbi.1000898 PMC2932675

[47] Feng X, Porporato A, Rodriguez-Iturbe I 2013 Changes in rainfall seasonality in the tropics. Nat. Clim. Change 3, 811–815. (doi:10.1038/nclimate1907)

[48] Oluwole OSA 2015 Seasonal influenza epidemics and El Ninos. Front. Pub. Health 3, 1–11. (doi:10.3389/fpubh.2015.00250) 26618150 10.3389/fpubh.2015.00250 PMC4639839

