# Supplementary material for: Effects of complexity and seasonality on backward bifurcation in vector–host models
Source: R Soc Open Sci. 2018 Feb 28;5(2):171971. doi: 10.1098/rsos.171971 (PMC5830785; doi:10.1098/rsos.171971)
Supplement: Basic Reproductive ratio [file rsos171971supp1.docx]

## Effects of complexity and seasonality on backward bifurcation in vector-host models

S. Bilal and E. Michael
(Department of Biological Sciences, University of Notre Dame, IN 46545, USA)

## **Appendix**.

The supporting information contains the following:

1. Seasonality.
2. The next generation matrix approach to calculate $R_{0}$.
3. The monodromy matrix method for $R_{0}(\varepsilon)$.
4. The Survival function approach for $R_{0}$.
5. The Formula for $R_{0}(\varepsilon)$ using Fourier-series.

### The next generation and monodromy matrices approach for $\boldsymbol{R}_{\boldsymbol{0}}$

In the absence of seasonality the number of secondary infections as a result of a single infectious host/vector ($R_{0}$) is given by the spectral radius of the next generation matrix $=FV^{-1}$ (1), where elements of $F$ and $V$ are given by

|  | $F_{ij}=\frac{\partial\mathcal{F}_{i}}{\partial x_{j}}$ $V_{ij}=\frac{\partial\mathcal{V}_{i}}{\partial x_{j}}$ |  |
| --- | --- | --- |

with $i,j=1,\ldots,m$ and each matrix is evaluated at the disease free equilibrium (DFE). The matrices $F$ and $V$ for $SEIRS-M_{S}M_{E}M_{I}$ model are obtained from Eq. (2) and (4) (see main text) by noting that it has four infected compartments ($E_{1},I_{1},M_{E},M_{I}$):

|  | $SEIRS-M_{S}{M_{E}M}_{I}:\left\{ \begin{aligned} F=\left[ \begin{matrix} 0 & 0 & 0 & b_{1}\beta_{H1} \\ 0 & 0 & 0 & 0 \\ 0 & \frac{b_{1}\beta_{1}\Pi_{M}\mu_{H1}}{\Pi_{H1}\mu_{M}} & 0 & 0 \\ 0 & 0 & 0 & 0 \end{matrix} \right] \\ V=\left[ \begin{matrix} Q_{1} & 0 & 0 & 0 \\ -\sigma_{H1} & Q_{2} & 0 & 0 \\ 0 & 0 & Q_{3} & 0 \\ 0 & 0 & -\sigma_{M} & Q_{4} \end{matrix} \right] \end{aligned} \right.$ | (1) |
| --- | --- | --- |

Seasonality is introduced in the system through the mosquito/vector death rate. We account for this by introducing a modulation of the death rate as follows:

|  | $\Pi\left( t \right)=\Pi_{M0}\left( 1+\varepsilon S(\omega_{0},\omega_{1},\ldots,\omega_{i},t) \right),$ |  |
| --- | --- | --- |

the parameter $\Pi_{M0}$ is the mean or unmodulated death rate of the vector and $\omega_{i}$ are the frequencies in the order $\omega_{0}<\omega_{1}<\ldots<\omega_{i}$ which implies that overall period of the system is given by $\omega_{i}$. The function $S\left( \omega_{0},\omega_{1},\ldots,\omega_{i},t \right)=s_{0}\left( \omega_{0},t \right)+\sum_{j=1}^{l} \varepsilon_{j}s_{j}\left( \omega_{j},t \right)$determines the nature of modulation ranging from, periodic to quasiperiodic depending on the relationship between the frequencies . Here we put for *j*=1,…,*l* and write . Thus ε is the strength of seasonality that determines the amplitude of seasonal variation in the death rate. A simple sinusoidal function $s_{0}\left( \omega_{0},t \right)=\sin2n\pi t/365$ with $\omega_{0}=365/n$ gives a $\frac{1}{n}$ year periodic birth rate (2):

|  | $\Pi\left( t \right)=\Pi_{M0}\left( 1+\varepsilon S(\omega_{0},\omega_{1},\ldots,\omega_{i},t) \right)$ | (2) |
| --- | --- | --- |

To keep $\Pi_{M}(t)\geq0$ we must have $0\leq\varepsilon\leq1$. The reproductive ratio is now time–dependent and is given by $R_{0}(t)$, however the average $R_{0}$ governing the *global stability* of DFE is calculated using Floquet theory [42, 43]. In this approach the number of secondary infections produced by a single infectious introduced at previous time *s* is given by the linear operator

|  | $\left( L\varphi\right)\left( t \right)=\int_{-\infty}^{t} Y\left( t,s \right)F\left( s \right)\varphi\left( s \right)ds$ | (3) |
| --- | --- | --- |

The spectral radius of the operator *L* is the basic reproductive ratio (3,4). Where $Y$ is the monodromy matrix associated with the equation for evolution of disease free equilibrium

|  | $\frac{dy}{dt}=-Vy$ | (4) |
| --- | --- | --- |

It was shown in (3) that the spectral radius of $L$ coincides with that value of parameter $\lambda$ in the following equation for which its monodromy matrix $W(t=T,\lambda)$ (where $T=\frac{2\pi}{\Omega}$ is the period of oscillations, $\Omega$ is the oscillation frequency) has largest eigenvalue equal to one (Theorem 2.1 in (3)):

|  | $\frac{dw}{dt}=\left[ -V\left( t \right)+\frac{F(t)}{\lambda} \right]w$ | (5) |
| --- | --- | --- |

that is the root $\lambda_{0}$ of the equation $\rho\left( W(T,\lambda) \right)=1$ is equal to the spectral radius of the operator *L*. Since we know the form of the matrices *V* and *F* for the $SEIRS-M_{S}M_{E}M_{I}$ model Eq. (1), we employ the bisection method to obtain ${R_{0}=\lambda}_{0}$ from Eq. (5). A bifurcation diagram generated using the linear operator method is shown in Fig. 1 below.


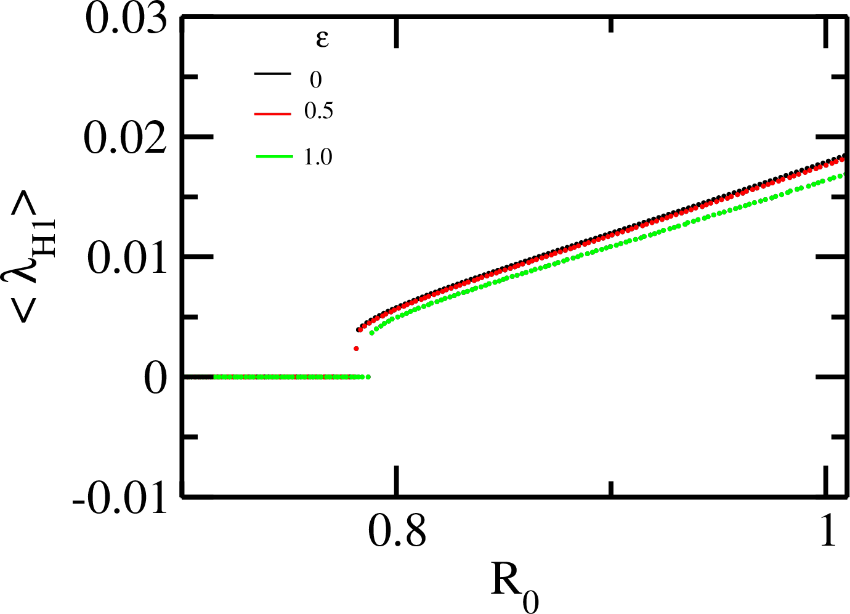

 Figure 1: Bifurcation diagram, showing only stable branches of $\left\langle\lambda_{H1} \right\rangle$ (time–averaged force of infection) corresponding to endemic (upper branches) and disease free (horizontal lines) equilibria, as a function of $R_{0}$. Colors for different ε are labeled in the figure. The stable orbits and $R_{0}$ were obtained by integrating Eq. (2) (see main text) and Eq. (5) numerically. Even though no approximations for $\Pi_{M}$ and $R_{0}$ were made, we observe that the backward bifurcation region decreases by increasing $\varepsilon$, (cf. Fig. 6 main text). The decrease in backward bifurcation region can be seen as a result of reduced the force of infection $\lambda_{H1}$.

### The Survival function and Fourier series approach

The survival function method $R_{0}$ is given by the simple formula

|  | $R_{0}=\left( number of infected hosts \right)\times\left( number of infected mosquitoes \right)$ |  |
| --- | --- | --- |
|  | $R_{0}=\left( \frac{b_{1}\beta_{H1}\sigma_{M}}{(\sigma_{M}+\mu_{M})(\mu_{M}+d_{M})} \right)\times\left( \frac{b\beta\mu\sigma_{H1}\Pi_{M}}{\Pi_{H1}\mu_{M}(\tau_{H1}+\mu_{H1}+d_{H1})(\sigma_{H1}+\mu_{H1})} \right).$ | (6) |

Using a slightly different definition of the Next Generation Operator

|  | $K=\left[ \begin{matrix} 0 & 0 & 0 & b_{1}\beta_{H1}e^{-Q_{4}t} \\ \sigma_{H1}e^{-Q_{1}t} & 0 & 0 & 0 \\ 0 & \frac{b_{1}\beta_{1}\Pi_{M}\mu_{H1}}{\Pi_{H1}\mu_{M}}e^{-Q_{2}t} & 0 & 0 \\ 0 & 0 & \sigma_{H1}e^{-Q_{3}t} & 0 \end{matrix} \right]$ | (7) |
| --- | --- | --- |

$R_{0}(\varepsilon)$ (in the limit of small $\varepsilon$) is obtained using a Fourier expansion method (see (5) for details):

|  | $R_{0}\left( \varepsilon\right)=G_{0}+\frac{\varepsilon^{2}}{2}\mathfrak{R}\left( \frac{G_{0}G_{1}}{G_{0}-G_{1}} \right)$ | (8) |
| --- | --- | --- |
|  | $G_{j}=\frac{b_{1}\beta_{1}\beta_{H1}\mu_{H1}\sigma_{H1}\sigma_{M}\Pi_{M0}}{\Pi_{H1}\mu_{M}(Q_{1}+ji\Omega)(Q_{2}+ji\Omega)(Q_{3}+ji\Omega)(Q_{4}+ji\Omega)}$ | (9) |

where $\mathfrak{R}$ represents real part of the complex number in the parenthesis. The expression for $A$ and $B$ in Eq. (21) (see main text), are then obtained as follows. Set

|  | $a_{1}=\Pi_{H1}\left( \mu_{H1}+\alpha_{H1} \right)Q_{1}Q_{2}Q_{3}Q_{4}$ $a_{2}=b_{1}\beta_{1}(\eta_{H1}Q_{2}+\sigma_{H1})\left( \mu_{H1}+\alpha_{H1} \right)$ $a_{3}=\left( \mu_{H1}+\alpha_{H1} \right)\sigma_{H1}+\tau_{H1}{\sigma_{H1}+Q}_{2}\left( \mu_{H1}+\alpha_{H1} \right)$ $a_{4}=\frac{Q_{1}Q_{2}\left( \mu_{H1}+\alpha_{H1} \right)-\alpha_{H1}\tau_{H1}\sigma_{H1}}{\mu_{H1}}$ $a_{5}=4a_{0}{{Q_{1}}^{2}{Q_{2}}^{2}Q_{3}Q_{4}\Pi}_{H1}\left( \mu_{H1}+\alpha_{H1} \right)^{2}\mu_{M}$ $h=a_{1}a_{2}+2\mu_{M}a_{1}a_{3}$ $A=\frac{a_{5}}{{\mu_{M}}^{2}{a_{1}}^{2}{a_{4}}^{2}}-\frac{2h}{\mu_{M}a_{1}a_{4}}$ $B=\frac{4(h^{2}-a_{5})}{{\mu_{M}}^{2}{a_{4}}^{2}{a_{1}}^{2}}$ |  |
| --- | --- | --- |

then the condition ${{b_{0}}^{2}-4a_{0}c}_{0}=0$, where $a_{0},b_{0},c_{0}$ are given by Eq. (17) –(19) (see main text), leads to the threshold $R_{c}$ given in Eq. (21) (see main text).

(1) Van den Driessche P, Watmough J. Reproduction numbers and sub-threshold endemic equilibria for compartmental models of disease transmission. Math Biosci 2002;180(1):29-48.

(2) Keeling MJ, Rohani P. Modeling infectious diseases in humans and animals. : Princeton University Press; 2008.

(3) Wang W, Zhao X. Threshold dynamics for compartmental epidemic models in periodic environments. Journal of Dynamics and Differential Equations 2008;20(3):699-717.

(4) Christopher David Mitchell. Reproductive numbers for periodic epidemic systems; 2016.

(5) Bacӓr N. Approximation of the basic reproduction number R_0_ for vector-borne diseases with a periodic vector population. Bull Math Biol 2007;69(3):1067-1091.
